# Lifestyle and dietary factors, iron status and one-carbon metabolism polymorphisms in a sample of Italian women and men attending a Transfusion Medicine Unit: a cross-sectional study

**DOI:** 10.1017/S0007114522003245

**Published:** 2023-07-14

**Authors:** Renata Bortolus, Francesca Filippini, Francesca Chiaffarino, Silvia Udali, Marianna Rinaldi, Giorgio Gandini, Martina Montagnana, Giuseppe Lippi, Oliviero Olivieri, Fabio Parazzini, Simonetta Friso

**Affiliations:** 1 Office for Research Promotion, Verona University Hospital, Verona, Italy; 2 Gynaecology Unit, Fondazione Istituto di Ricovero e Cura a Carattere Scientifico (IRCCS) Ca’ Granda Ospedale Maggiore Policlinico, Milan, Italy; 3 Unit of Internal Medicine B, Department of Medicine, University of Verona School of Medicine, Verona, Italy; 4 Unit of Transfusion Medicine, Verona University Hospital, Verona, Italy; 5 Section of Clinical Biochemistry, Department of Neurosciences, Biomedicine and Movement Sciences, University of Verona School of Medicine, Verona, Italy; 6 Department of Clinical Sciences and Community Health, Università degli Studi di Milano, Milan, Italy

**Keywords:** Lifestyle and dietary factors, Fe status, One-carbon metabolism polymorphisms, Fe

## Abstract

Iron (Fe) status among healthy male and female blood donors, aged 18–65 years, is estimated. General characteristics and lifestyle factors, dietary habits and major one-carbon metabolism-related polymorphisms were also investigated. An explorative cross-sectional study design was used to examine a sample of blood donors attending the Transfusion Medicine Unit of the Verona University Hospital, Italy. From April 2016 to May 2018, 499 subjects were enrolled (255 men, 244 women, 155 of whom of childbearing age). Major clinical characteristics including lifestyle, dietary habits and Fe status were analysed. The *MTHFR* 677C > T, *cSHMT* 1420C > T, *DHFR* 19bp ins/del and *RFC1* 80G > A polymorphisms were also assayed. Mean plasma concentrations of Fe and ferritin were 16·6 µmol/l (95 % CI 16·0, 17·2) and 33·8 µg/l (95 % CI 31·5, 36·2), respectively. Adequate plasma Fe concentrations (> 10·74 µmol/l) were detected in 84·3 % and adequate ferritin concentrations (20–200 µg/l) was found in 72·5 % of the whole cohort. Among the folate-related polymorphisms analysed, carriers of the *DHFR* 19bp del/del mutant allele showed lower ferritin concentration when compared with *DHFR* 19bp ins/del genotypes. In a sample of Italian healthy blood donors, adequate plasma concentrations of Fe and ferritin were reached in a large proportion of subjects. The relationship of Fe status with lifestyle factors and folate-related polymorphisms requires more investigation to clarify further gene–nutrient interactions between folate and Fe metabolism.

Fe status is critical to define the health status of a population^(1)^, and this is also due to its major role in influencing the risk of chronic diseases^(2,3)^. The knowledge of precise blood concentrations of Fe and ferritin is, therefore, a main issue to design public health interventions on target populations^(4)^.

Considering the relevance of defining Fe and ferritin concentrations for a clear definition of dietary policies, along with the possible indication of supplementation, measuring Fe status in a sample of healthy men and women is essential. Only few data, however, are so far available from studies conducted in Italy on the average serum levels of Fe and ferritin in the general population^(5–7)^.

Furthermore, an interesting link has been highlighted between folate and Fe, whereby perturbations in Fe status and metabolism may impair folate metabolism mainly through the function of cytosolic serine hydroxymethyltransferase (*cSHMT*), an enzyme which catalyses either nucleotide synthesis or methyl group provision within the folate-related one-carbon metabolism^(8–10)^. The expression of *cSHMT* is increased by elevated heavy chain ferritin concentrations, independent of enhanced Fe availability, suggesting that *cSHMT* expression influenced also by the functional 1420C > T polymorphism may respond to heavy chain ferritin (HCF)-induced chelation of the regulatory Fe pool^(10)^.

Based on the knowledge that gene–nutrient interactions occur by the relationship through which genetic signatures influence nutrient availability together with mutation selection in a population^(11)^, and considering the assumption that genetic signatures may contribute to differences in nutritional status and eventually define different human nutritional requirement based on genetic features of a certain population^(11)^, we chose to evaluate all the major functional DNA polymorphisms within folate metabolism.

The link between one-carbon and Fe metabolism and the allele frequency for common folate-related polymorphisms is yet mostly unknown in the general population. Indeed, the study of a potential association between Fe status and one-carbon metabolism polymorphisms, including *cSHMT* 1420C > T, dihydrofolate reductase (*DHFR*) 19bp ins/del, Rmethylenetetrahydrofolate reductase (*MTHFR*) 677C > T and reduced folate carrier (*RFC1*) 80G > A, shall be considered to explore promising yet unknown gene–nutrient interactions and molecular mechanism within folate and Fe metabolism regulating disease development and prevention^(12,13)^.

The aim of the study was to define, in a sample of men and women aged 18–65 years attending a Transfusion Medicine Unit, the lifestyle factors and the main determinants of Fe status by assessment of plasma Fe and ferritin concentrations, together with the assessment of major one-carbon-related polymorphisms including *cSHMT* 1420C > T, *MTHFR* 677C > T, *DHFR* 19bp ins/del and *RFC1* 80G > A.

## Subjects and methods

### Ethical standards

The study was approved by the Ethical Committee of the Verona University Hospital. Eligible subjects were informed about the objectives and procedures of the study. All participants provided written informed consent before enrolment. The procedures used were in accordance with the ethical standards of the responsible institutional or regional committee on human experimentation or in accordance with the Helsinki Declaration of 1975 as revised in 1983.

### Study design and participants

The study design has already been published elsewhere^(14)^. Briefly, from April 2016 to May 2018, 551 healthy blood donors, consecutively attending the Transfusion Medicine Unit of the Verona University Hospital (Italy), were enrolled in this cross-sectional study, 538 of whom (97·6 %) accepted. Overall, 499 subjects were finally included in the study (255 men, 244 women: 155 of childbearing age 18–44 years).

During the visit to be enrolled for the blood donation, each eligible subject, after detailed explanation of the study design, was invited to participate. After giving written informed consent, each participant was interviewed about his/her general characteristics, medical history and current therapy. Lifestyle, education as a recognised parameter of lifestyle and dietary behaviour, diet and dietary habits including alcohol and smoking habits, fruits and vegetables consumption were also recorded. For fruits and vegetables, one portion was defined as 150 and 100 g, respectively.

### Laboratory parameters

Venous whole blood samples were collected after an overnight fasting into Vacutainer® tubes either containing EDTA or lithium/heparin as anticoagulants, to measure biochemical variables and for extracting DNA from peripheral blood mononuclear cells.

After centrifugation at 1500 *g* for 10 min at room temperature, lithium heparin plasma was separated, stored in aliquots and kept frozen at −70°C until measurement. Fe was determined by the routine method used in the local laboratory (Roche Diagnostics). Ferritin concentration was measured with an automated chemiluminescence method, on Roche Cobas e801 (Roche Diagnostics). DNA was extracted from peripheral blood mononuclear cells by Wizard Genomic DNA Purification Kit (Promega Corporation). Genotyping for one-carbon-related polymorphisms (*MTHFR* 677C > T, *cSHMT* 1420C > T, *DHFR* 19bp ins/del, *RFC1* 80G > A) was analysed by different methods as previously described^(14)^.

A plasma Fe concentration > 10·74 μmol/l and ferritin range values 20–200 µg/l were considered as adequate concentrations^(15)^.

### Statistical analysis

Data were collected in a specific database after a review for completeness, consistency, and plausibility. This study included 499 subjects. The original sample size was computed considering as the primary objective of the study the frequency of adequate plasma folate concentrations (> 15 nmol/l)^(14)^. Considering the end point of the present analysis (i.e. the frequency of adequate Fe status) based on a post hoc computation, we were able to obtain estimates of adequate plasma Fe concentrations with narrow 95 % CI. For example, considering separately men and women, the expected 95 % CI were, respectively, 74·3–84·8 if the adequate plasma Fe concentrations were 80 % or 85·7–93·6 if the values were 90 % (sample size 244 for each group).

Continuous variables were calculated by mean values and standard deviations, while logarithmic transformation was used for not-normally distributed variables, for which geometric means and CI were used, as appropriate. Categorical variables were presented by calculating absolute frequency and percentage. The 95 % CI of the mean and proportion were provided to assess the precision of estimates. Genetic data were analysed to evaluate the frequency of each genotype in the population studied after evaluating the Hardy Weinberg equilibrium. Categorical variables were compared using the Pearson or Mantel–Haenszel *χ* ^2^, as appropriate. Continuous variables were analysed using ANOVA, after logarithmic transformation if needed, or Kruskal–Wallis test when appropriate. We considered a two-tailed *P* value of < 0·05 to be significant. OR for inadequate status of Fe and ferritin according to socio-demographic and general characteristics were computed. To take into account potential confounding factors, we used unconditional multiple logistic regression, with maximum likelihood fitting including in the model terms for sex, age, education, BMI, smoking, alcohol drinking, fruits and vegetables consumption and physical activity. All the analyses were performed using the SAS software, version 9.4 (SAS Institute, Inc).

## Results

### Characteristics of the study population

Socio-demographic and general characteristics of the study population according to age and sex are shown in Table 1.

**Table 1.** Socio-demographic and general characteristics of the subjects according to age and sex (Numbers and percentages)

	Total		Females < 45 years		Females ≥ 45 years			Males < 45 years		Males ≥ 45 years		
	*n*	%	*n*	%	*n*	%	*P* *	*n*	%	*n*	%	*P* *
Age (years)												
≤ 34	124	24·9	94	60·7				30	29·4			
35–44	133	26·7	61	39·4				72	70·6			
45–54	164	32·9			73	82·0				91	59·5	
≥ 55	78	15·6			16	18·0				62	40·5	
Education (years)							0·0002					< 0·0001
≤ 8	80	16·0	9	5·8	21	23·6		6	5·9	44	28·8	
9–15	301	60·3	91	58·7	46	51·7		72	70·6	92	60·1	
≥ 16	118	23·7	55	35·5	22	24·7		24	23·5	17	11·1	
BMI (kg/m^2^)							0·0536					0·1909
Underweight	12	2·4	10	6·5	1	1·1		0		1	0·7	
Normal weight	328	65·7	111	71·6	74	83·2		62	60·8	81	52·9	
Overweight	127	25·5	25	16·1	13	14·6		35	34·3	54	35·3	
Obesity	32	6·4	9	5·8	1	1·1		5	4·9	17	11·1	
Current smoking							0·5678					0·003
No	424	85·0	128	82·6	76	85·4		80	78·4	140	91·5	
Yes	75	15·0	27	17·4	13	14·6		22	21·6	13	8·5	
Alcohol intake							0·5218					0·03
No	120	24·1	58	37·4	37	41·6		5	4·9	20	13·1	
Yes	379	76·0	97	62·6	52	58·4		97	95·1	133	86·9	
Fruits							0·0709					0·1615
< 1 portion/d	138	27·7	46	29·7	16	18·0		37	36·3	39	25·5	
1–2 portion/d	278	55·7	88	56·8	54	60·7		48	47·1	88	57·5	
> 2 portion/day	83	16·3	21	13·6	19	21·4		17	16·7	26	17·0	
Vegetables							0·0083					0·7859
< 1 portion/d	55	11·0	15	9·7	1	1·1		15	14·7	24	15·7	
1–2 portion/day	370	74·2	101	65·2	72	80·9		78	76·5	119	77·8	
> 2 portion/d	74	14·8	39	25·2	16	18·0		9	8·8	10	6·5	
Physical activity							0·8962					0·7895
< 2 h/week	200	40·1	68	43·9	38	42·7		40	39·2	54	35·3	
2–5 h/week	197	39·5	66	42·6	37	41·6		37	36·3	57	37·3	
> 5 h/week	102	20·4	21	13·6	14	15·7		25	24·5	42	27·5	

* *P* values were obtained by *χ* ^2^ test for the comparison within each sex group, that is, among females and males, divided by age either < 45 years or ≥ 45 years. Underweight and normal weight were considered together.

### Biochemical parameters

The mean plasma Fe and ferritin concentrations were 16·6 (95 % CI 16·0, 17·2) µmol/l and 33·8 (95 % CI 31·5, 36·2) µg/l, respectively. Males had significantly higher plasma concentrations of Fe and ferritin than females, while women < 45 years had lower ferritin concentrations compared with those aged 45 years or older (Table 2).

**Table 2.** Mean plasma concentrations and prevalence of subjects with adequate Fe and ferritin according to sex and age (Mean values and 95 % confidence intervals; numbers and percentages)

	Total *n* 499		Females *n* 244		Males *n* 255		*P* *	Females < 45 years *n* 155		Females ≥ 45 years *n* 89		*P* *	Males < 45 years *n* 102		Males ≥ 45 years *n* 153		*P* *
	Mean	95 % CI	Mean	95 % CI	Mean	95 % CI		Mean	95 % CI	Mean	95 % CI		Mean	95 % CI	Mean	95 % CI	
Fe (µmol/l)	16·6	16·0, 17·2	15·4	14·6, 16·3	17·8	16·9, 18·7	0·0002	14·8	13·7, 16·0	16·6	15·4, 17·9	0·0545	17·9	16·5, 19·4	17·7	16·6, 18·8	0·817
> 10·74 μmol/l†																	
*n*	421		197		224		0·0289	118		79		0·0160	86		138		0·2225
%	84·3		80·7		87·8			76·1		88·8			85·2		90·2		
Ferritin (µg/l)	33·8	31·5, 36·2	26·6	24·2, 29·3	42·5	38·6, 46·7	< 0·0001	24·3	21·7, 27·2	31·1	26·3, 36·8	0·0130	41·3	35·2, 48·4	43·2	38·3, 48·8	0·6398
20–200 μg/l†																	
*n*	362		159		203		0·0003	96		63		0·1625	76		127		0·1308
%	72·5		65·1		79·6			61·9		70·8			75·3		83·0		

* *P* value ≤ 0·05 was considered statistically significant. *P* values were obtained by *χ* ^2^ test for the comparison within each sex group, that is, among females and males, divided by age either < 45 years or ≥ 45 years.

† Adequate concentrations.

Adequate Fe concentrations, defined as plasma levels > 10·74 µmol/l, were observed in 84·3 % of total blood donors, while adequate ferritin concentrations, defined as values ranging between 20 and 200 µg/l, were found in 72·5 % of total blood donors; 80·7 % females *v*. 87·8 % males displayed adequate Fe concentrations, while 65·1 % females *v*. 79·6 % males displayed adequate ferritin concentrations, with significant difference between sexes (Table 2). In females < 45 years of age, the prevalence of subjects with adequate Fe concentrations was significantly lower compared with females aged ≥ 45 years (Table 2).

In Table 3, intake of ≥ 3 portion/d of fruits and vegetables was associated with inadequate plasma Fe levels, while alcohol intake was associated with adequate plasma Fe concentrations. No relation was found between these determinants and ferritin concentrations. No significant differences emerged in Fe status according to other general characteristics and lifestyle factors.

**Table 3.** Odds ratios for inadequate status of Fe and ferritin according to general characteristics and lifestyle factors (Odds ratios and 95 % confidence intervals)

		Fe ≤ 10·74 μmol/l†		Ferritin < 20–> 200 µg /l†	
	*n*	OR	95 % CI*	OR	95 % CI*
Sex‡					
Female	255	1·06	0·57, 1·97	1·73	1·07, 2·81
Age (years)‡					
35–44	133	0·97	0·44, 2·15	1·18	0·61, 2·30
45–54	164	0·67	0·33, 1·38	1·01	0·31, 1·24
≥ 55	78	0·42	0·18, 1·00	0·62	0·31, 1·24
Education (years)‡					
9–15	301	1·55	0·65, 3·78	0·97	0·52, 1·81
≥ 16	118	1·27	0·48, 3·39	0·89	0·43, 1·84
BMI (kg/m^2^)‡					
Underweight	12	0·29	0·03, 2·45	0·96	0·27, 3·41
Overweight	127	1·14	0·61, 2·15	1·09	0·66, 1·80
Obesity	32	1·64	0·60, 4·51	0·68	0·26, 1·77
Current smoking‡					
Yes	75	1·42	0·72, 2·78	1·2	0·72, 2·18
Alcohol drinking‡					
Yes	379	0·38	0·22, 0·69	0·71	0·44, 1·15
Fruits and vegetables‡					
2 portion/d	232	1·16	0·61, 2·22	1·05	0·64, 1·70
≥ 3 portion/d	115	2·27	1·13, 4·58	1·00	0·56, 1·79
Physical activity‡					
2–5 h/week	197	0·73	0·41, 1·31	0·85	0·54, 1·34
> 5 h/week	102	1·00	0·50, 1·99	0·78	0·44, 1·41

* Multivariate estimates including in turn term for sex, age, education, BMI, smoking, alcohol drinking, fruits and vegetables intake, and physical activity.

† Inadequate concentrations.

‡ Reference categories: male (sex), ≤ 34 years (age), < 8 years (education), normal weight (BMI), no (current smoker), no (alcohol drinking), < 1 portion/d (fruits and vegetables), < 2 h/week (physical activity).

### One-carbon metabolism-related polymorphisms

The homozygous mutant allele frequencies were 0·21 for the *MTHFR* 677TT, 0·11 for the *cSHMT* 1420TT, 0·18 for the *DHFR* 19bp del/del and 0·20 for the *RFC1* 80AA. As for a possible association among plasma Fe and ferritin concentrations and *MTHFR* 677C > T, *cSHMT* 1420C > T and *RFC1* 80G > A polymorphisms, no relationship was detected. Carriers of the *DHFR* 19bp del/del genotype showed lower ferritin concentrations compared with the *DHFR* 19bp ins/del genotypes (29·5 (95 % CI 24·4, 35·6) µg/l *v*. 37·3 (95 % CI 33·5, 41·5) µg/l, *P* = 0·02). When data were analysed for the comparison of carriers of the mutant allele *DHFR* 19bp ins/del plus del/del genotypes *v*. the *DHFR* 19bp ins/ins, no differences were observed for plasma ferritin concentrations (34·9 (95 % CI 31·7, 38·3) µg/l *v*. 31·8 (95 % CI 28·5, 35·6) µg/l, respectively, *P* = 0·22).

## Discussion

The study provides information on lifestyle, dietary factors, Fe status and *MTHFR* 677C > T, *cSHMT* 1420C > T, *DHFR* 19bp ins/del, *RFC1* 80G > A genotypes in a sample of healthy Italian women and men aged 18–65 years, attending a Transfusion Medicine Unit in Northern Italy.

The study shows that adequate Fe status in terms of both plasma Fe and ferritin concentrations could be reached in a large proportion of this Italian sample of healthy blood donors. Moreover, intake of ≥ 3 portion/d of fruits and vegetables was found to be associated with inadequate plasma Fe concentrations unlike ferritin. Fe available in foodstuffs can be of heme and non-heme type. In animal-origin products, 40 % of the existing Fe is of heme type, and 60 % is non-heme, whereas plant-origin foodstuffs only contain non-heme Fe. Heme Fe is absorbed by 15–35 % in the gastrointestinal tract, whereas non-heme Fe presents lower absorption, between 2 and 20 %^(16)^. Non-heme-Fe absorption is strongly influenced by many inhibitory and enhancing factors in the diet, whereas heme-Fe absorption is very little affected by other dietary components. Furthermore, early studies with radioisotope labelled foods found that Fe from animal foods was better absorbed than that from plant foods^(17)^. While emphasising that fruit and vegetables are essential to the diet, this varying bioavailability can support our results.

Regarding the association between alcohol intake and adequate status of Fe but not ferritin, we should consider that Fe bioavailability is influenced by various dietary components that either enhance or inhibit its absorption. Alcohol-induced disorders of the Fe metabolism were investigated in animal models and clinical and epidemiological studies^(18,19)^. Furthermore, increases in indices of Fe stores, such as serum ferritin, have also been described in subjects drinking small amounts of alcohol compared with teetotallers^(20,21)^ and there is evidence that both Fe and alcohol can initiate the formation of free radicals and produce oxidative stress within the liver^(22–24)^. The relationship between alcohol intake and Fe stores is therefore of interest also among the general population; however, in evaluating this association it is important to consider the whole picture rather than relying on single test result.

Regarding Fe status in Italian population, Salvaggio *et al.* ^(5)^ studied 400 subjects, 200 men and 200 women, aged between 20 and 60 years, reporting that the frequency of Fe deficiency was increased in women of childbearing age. Overall, 13 % of women in the three younger age groups had low serum ferritin levels. Only 6 % of women aged over 50 years were instead found to be Fe deficient, according to other published studies^(25,26)^.

Most recently, a population-based study in primary care^(6)^ showed that the incidence rate of Fe-deficiency anaemia increased by 51·4 % over a nearly 10-year period in Italy, from 5·9 per 1000 person-years in 2002 to 8·93 per 1000 person-years in 2013 with an incidence rate in females that was almost 4-fold higher than in males. As for women in childbearing age, the present study reveals adequate levels of Fe and ferritin in 76·1 and 61·9 % women aged 18–44 years, respectively.

Considering the determinants of Fe status, our results confirm that alcohol intake was associated with adequate Fe status, as already shown in earlier investigations^(27,28)^, unlike ferritin^(29)^.

Moreover, higher consumption of fruits and vegetables (i.e. ≥ 3 portion/d) was associated with lower levels of Fe but not of ferritin, with evidence for the latter biomarker already reported by other studies^(30)^. Scarce and controversial information remains currently available on this association^(31–33)^. As for a possible association between markers of Fe status and *cSHMT* 1420C > T and the other common one-carbon metabolism polymorphisms analysed, no relationship was observed. Interestingly, however, carriers of the *DHFR* 19bp del/del genotype showed lower ferritin concentrations when compared with the *DHFR* 19bp ins/del genotypes. Although there is no evident explanation for this finding, a possible hypothesis is related to the loss of enzymatic function induced by the *DHFR* 19bp polymorphism, as it occurs in other models in which *DHFR* silence reduces the development of liver fibrosis by altering the crosstalk between hepatic stellate cells and macrophages. This is a crucial event underlying inflammation^(34)^, where ferritin is a marker of macrophage activation^(35)^.

Another hypothesis may be suggested through the mechanisms regulating the ferritin-mediated folate catabolism and turnover of differential folate compounds^(36)^ in which the DHFR enzyme may be involved as a key factor for the balance of the biochemical functions of folate degradation of labile forms of folate^(37)^.

Nonetheless, further specific studies would be needed to better explore this topic.

The study has some weaknesses and strengths. It is mostly based on a small and specific population, perhaps not representative of the Italian healthy adult population. We evaluated blood donors: these subjects are considered a healthier group, so the results expected in the general population could be different, though we overcome this potential bias by balancing the age and sex groups. Moreover, elderly people are not eligible for blood donation and, therefore, excluded. Finally, regarding determinants of Fe status, fruits and vegetables components of diet and level of alcohol intake were not investigated.

Among the strengths of this study, we consider the overall availability of data on lifestyle and dietary factors along with blood concentrations of Fe and ferritin in Italian population that may be helpful to design public health interventions on the target population, especially in subgroups of population with special needs. Furthermore, it is of interest the evaluation of the potential impact of common one-carbon-related genetic variants influencing Fe status.

### Conclusion

In conclusion, adequate concentrations of Fe and ferritin were reached in a large proportion of an Italian sample of healthy blood donors. The relation of Fe status with lifestyle factors and the one-carbon polymorphisms investigated requires further research to better clarify possible further gene–nutrient interactions involved in folate and Fe metabolism.
